# Protein phosphatase Mg2+/Mn2+ dependent 1F promotes smoking-induced breast cancer by inactivating phosphorylated-p53-induced signals

**DOI:** 10.18632/oncotarget.12717

**Published:** 2016-10-18

**Authors:** Shih-Hsin Tu, Yin-Ching Lin, Chi-Cheng Huang, Po-Sheng Yang, Hui-Wen Chang, Chien-Hsi Chang, Chih-Hsiung Wu, Li-Ching Chen, Yuan-Soon Ho

**Affiliations:** ^1^ Department of Surgery, School of Medicine, College of Medicine, Taipei Medical University, Taipei, Taiwan; ^2^ Breast Medical Center, Taipei Medical University Hospital, Taipei, Taiwan; ^3^ Taipei Cancer Center, Taipei Medical University, Taipei, Taiwan; ^4^ Graduate Institute of Medical Sciences, College of Medicine, Taipei Medical University, Taipei, Taiwan; ^5^ School of Medicine, College of Medicine, Fu-Jen Catholic University, New Taipei City, Taiwan; ^6^ Breast Center, Cathay General Hospital, Taipei, Taiwan; ^7^ Department of Surgery, Mackay Memorial Hospital, Taipei, Taiwan; ^8^ Department of Medicine, Mackay Medical College, New Taipei City, Taiwan; ^9^ Department of Laboratory Medicine, Taipei Medical University Hospital, Taipei, Taiwan; ^10^ Department of Surgery, En Chu Kong Hospital, New Taipei City, Taiwan; ^11^ Graduate Institute of Clinical Medicine, College of Medicine, Taipei Medical University, Taipei, Taiwan; ^12^ Comprehensive Cancer Center of Taipei Medical University, Taipei, Taiwan; ^13^ School of Medical Laboratory Science and Biotechnology, College of Medical Science and Technology, Taipei Medical University, Taipei, Taiwan

**Keywords:** protein phosphatase Mg^2+^/Mn^2+^ dependent 1F, breast cancer, smoking, α9-nicotinic acetylcholine receptor, p53

## Abstract

We previously demonstrated that the activation of α9-nicotinic acetylcholine receptor (α9-nAchR) signaling by smoking promotes breast cancer formation. To investigate the downstream signaling molecules involved in α9-nAChR-induced breast tumorigenesis, we used real-time polymerase chain reactions and Western blotting to assess expression of protein phosphatase Mg^2+^/Mn^2+^ dependent 1F (PPM1F), a Ser/Thr protein phosphatase, in human breast cancer samples (n=167). Additionally, stable *PPM1F*-knockdown and -overexpressing cell lines were established to evaluate the function of PPM1F. The phosphatase activity of PPM1F in nicotine-treated cells was assessed through Western blotting, confocal microscopy, and fluorescence resonance energy transfer. Higher levels of *PPM1F* were detected in the breast cancer tissues of heavy smokers (n=7, 12.8-fold) greater than of non-smokers (n= 28, 6.3-fold) (**p=0.01). *In vitro*, nicotine induced PPM1F expression, whereas α9-nAChR knockdown reduced the protein expression of PPM1F. A series of biochemical experiments using nicotine-treated cells suggested that the dephosphorylation of p53 (Ser-20) and BAX (Ser-184) by PPM1F is a critical posttranslational modification, as observed in breast cancer patients who were heavy smokers. These observations indicate that PPM1F may be a mediator downstream of α9-nAChR that activates smoking-induced carcinogenic signals. Thus, PPM1F expression could be used for prognostic diagnosis or inhibited for cancer prevention and therapy.

## INTRODUCTION

Breast cancers are caused by defects in cell cycle checkpoints that allow damaged DNA to go unrepaired [1]. Cigarette smoke contains compounds that may damage DNA, and the repair of this damage may be impaired in women with germline mutations in BRCA1 or BRCA2 [2]. Deficiencies in DNA repair have been associated with breast cancer risk. A previous study demonstrated that the DNA damage response and repair capacity, was consistently associated with the risk of tobacco-related breast cancers [3].

One of the major checkpoints in mammalian cells is the G1/S checkpoint, regulated by p53, which responds to exposure to DNA-damaging agents [4]. Many studies have been conducted to determine whether p53 gene polymorphisms modify the association between smoking and breast cancer. The results of these epidemiological studies have been consistent with previous evidence of exposure-specific p53 mutations in breast tumors from current and former smokers, suggesting that smoking may be an important factor in breast cancer etiology [5]. The discovery of smoking-specific DNA adducts and p53 gene mutations in the breast tissue of smokers also supports the biological plausibility of a positive association between cigarette smoking and breast cancer [6–8].

In cancer therapy, DNA-damaging agents have been shown to kill tumor cells by promoting p53-induced apoptosis, primarily by reducing the degradation of p53 [9, 10], and, to a lesser extent, by increasing the translation efficiency of *p53* mRNA [11]. The protein stability of p53 depends on the time required for its intracellular degradation. It has been reported that MDM2, an intracellular protein, binds to p53 [12] and exports it from the nucleus for ubiquitin-dependent proteolysis [13]. In response to DNA-damaging agents, posttranslational modifications such as the phosphorylation of Ser-15 [14, 15], Thr-18 [16], and Ser-20 [17] weaken the binding between p53 and MDM2. This is thought to stabilize the newly synthesized p53 protein by preventing its MDM2-dependent degradation [18].

Accordingly, understanding how p53 is inactivated by smoking could facilitate efforts to prevent and treat of cancer. Previous animal studies demonstrated that the protein levels of phosphorylated p53, total p53, and p53-regulated genes (p21 [WAF1/CIP1] and BAX-1) increased substantially in the lungs of ferrets subjected to long-term (9 weeks to 6 months) cigarette smoke exposure [19, 20]. However, such results do not explain how environmental factors (such as smoke) induce breast cancer cell transformation through the accumulation of p53 protein. We previously demonstrated that extremely low-dose (8 nM) nicotine could saturate the α9-nicotinic acetylcholine receptor (α9-nAChR) expressed on breast cancer cells [21]. Such results imply that the activation of receptor-induced signaling is important for smoking-induced breast cancer formation [22]. Based on these observations, we propose that another molecule could be important for the inactivation of p53 protein during smoking-induced breast cancer formation.

PPM1F (also call POPX2) is a serine/threonine phosphatase belonging to the protein phosphatase 2C family [23] that is overexpressed in invasive breast cancer cells [24]. MicroRNA-200c, which was previously reported to suppress the epithelial-mesenchymal transition [25, 26], was recently demonstrated to do so mainly by repressing the migration and invasion of breast cancer cells by downregulating *PPM1F* [26]. PPM1F promotes cancer cell migration and metastasis [27], and silencing of this gene reduces cell motility and invasiveness [28], but little is known about how PPM1F produces these effects. In this study, higher levels of *PPM1F* were detected in breast cancer tissue from heavy smokers (12.8-fold) with advanced-stage disease (stages 3-4) than in non-smokers with advanced-stage disease (6.3-fold). An *in vitro* study also demonstrated that the overexpression of *PPM1F* significantly reduced the level of phosphorylated p53 (Ser-20) in nicotine-treated breast cancer cells. We suggest that PPM1F is a gatekeeping protein that suppresses the activity of p53 and its downstream genes and thus promotes smoking-induced breast cancer.

## RESULTS

### *PPM1F* mRNA was highly expressed in human breast tumor tissues

**Figure 1:**
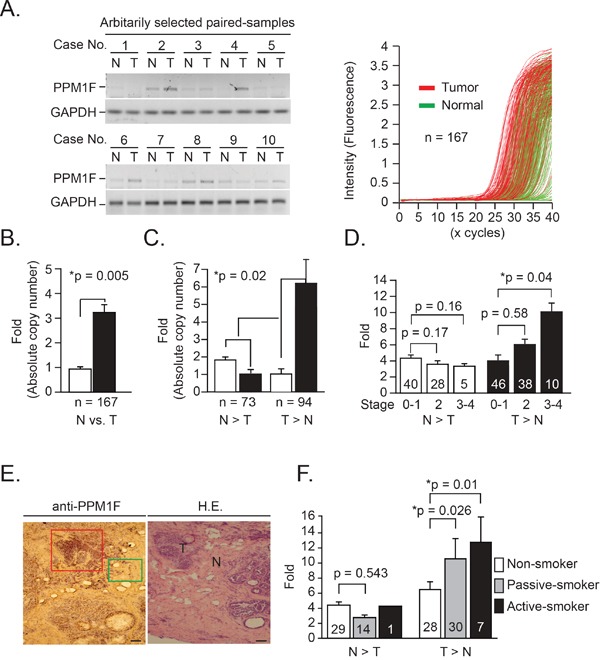
*PPM1F* mRNA and protein levels in human breast tumor tissues **A.** Left, PPM1F protein expression was determined by Western blotting in paired breast tumor tissues (10 cases) randomly selected from 167 cases. Right, *PPM1F* mRNA expression profiles in paired human breast tumor (red lines) and normal (green lines) tissues (n = 167) were evaluated by real-time PCR. **B.** *PPM1F* mRNA levels were determined in paired samples (n = 167) by real-time PCR; copy numbers (× 10^3^ per μg mRNA). Results were calculated from the mean real-time PCR data; error bars indicate the 95% confidence interval (C.I.). **C.** The *PPM1F* mRNA levels detected by PCR in paired samples from the 167 cases were divided into the T > N (n = 94) and N > T (n = 73) groups. The fold changes in mRNA expression are detected by real-time PCR and calculated by comparison in each tumor/normal paired samples. **D.** The mean data for the *PPM1F* mRNA levels detected by PCR in paired samples from the 167 cases were divided into four subgroups based on the clinical staging criteria recommended by the American Joint Committee on Cancer. Error bars indicate the 95% C.I. The data were analyzed with an overall nonparametric test (Kruskal-Wallis test), and multiple comparisons were performed with the Mann-Whitney test. All the *p*-values are two-sided. **E.** Human breast tumor tissues were cut into 7-μm serial sections and stained with antibodies specific to human PPM1F antigen. H.E., hematoxylin and eosin stain. Scale bar = 200 μm. **F.** The mean data for the *PPM1F* mRNA levels detected by PCR in paired samples from the 167 cases were divided into three subgroups based on the patients' clinical smoking history. Error bars indicate the 95% C.I. The data were analyzed with an overall nonparametric test (Kruskal-Wallis test), and multiple comparisons were performed with the Mann-Whitney test. All the *p*-values are two-sided.

Ten paired samples were arbitrarily selected from breast cancer patients (n = 167), and PPM1F protein levels were determined by immunoblotting analysis. Higher levels of PPM1F were detected in tumor tissues (T) than in normal (N) tissues (Figure 1A, left panel). Additionally, *PPM1F* mRNA levels in paired samples were examined by real-time RT-PCR (Figure 1A, right panel, n = 167). When all cases were averaged (n = 167), the average *PPM1F* copy number (x 10^3^/μg mRNA) in paired tumor tissues was 3.23-fold greater than in normal tissues (Figure 1B, bar 1 vs. bar 2, *p = 0.005). As shown in Figure 1C, all cases were further divided into two groups based on *PPM1F* mRNA levels. In the normal > tumor (denoted as N > T) group, the mean *PPM1F* mRNA level in the normal tissue was less than 2-fold greater (Figure 1C, bar 1 vs. bar 2, *p = 0.01). In the tumor > normal (denoted as T > N) group, the mean *PPM1F* mRNA level in the tumor tissue was 6.3-fold greater (Figure 1C, bar 3 vs. bar 4, *p = 0.001). A significant difference in *PPM1F* expression was detected between these two groups (*p = 0.02).

### PPM1F protein expression was higher in advanced-stage breast tumor tissues

As shown in Figure 1D, elevated *PPM1F* mRNA levels were detected preferentially in advanced-stage tumors rather than early-stage tumors (T>N group, stages 0-1 vs. stages 3-4, *p = 0.04; Table 1). PPM1F protein expression was also determined through IHC staining of frozen tumor sections (Figure 1E), PPM1F expression was greater in tumor samples (indicated with a red box) than in the adjacent normal tissues (indicated with a green box).

**Table 1:** Demographic evaluation of clinical criteria and changes in PPM1F1 mRNA expression fold ratios of tumor/normal paired samples

Factors	PPM1F1 N>T			PPM1F1 T>N		
	(n)	^§^mean ± SEM	*p* Value	(n)	^§^mean ± SEM	*p* Value
Age			0.82			0.29
<50yr	39	1.5 ± 0.2		52	4.9 ± 0.4	
≥50yr	34	1.4 ± 0.2		42	4.8 ± 0.5	
Size of tumor			0.12			0.01*
T1	32	2.6 ± 0.3		35	3.6 ± 0.5	
T2	24	2.5 ± 0.3		38	4.6 ± 0.8	
T3	11	0.4 ± 0.2		13	6.9 ± 1.8	
T4	6	4.5 ± 2.3		8	0.6 ± 0.3	
Nodal status			0.5			0.8
N0	23	1.5 ± 0.2		42	3.5 ± 0.5	
N1	25	1.2 ± 0.2		29	4.2 ± 0.8	
N2	12	1.2 ± 0.3		13	2.8 ± 0.6	
N3	13	2.0 ± 0.7		10	4.0 ± 1.1	
Stage			0.15			0.02*
Tis&I	40	4.2 ± 1.3		46	4.0 ± 0.3	
II	28	3.8 ± 1.1		38	5.9 ± 0.4	
III&IV	5	3.7 ± 1.2		10	10.1 ± 0.7	
Metastasis			0.12			0.01*
No	70	1.7 ± 0.3		70	1.7 ± 0.3	
Yes	1	0.8 ± 0.6		20	2.5 ± 1.8	
Smoke status			0.59			*0.02
Non-	29	4.3 ± 0.8		28	6.3 ± 1.5	
Passive-	14	2.9 ± 0.6		30	10.3 ± 2.4	
Current-	1	4.2 ± 1.8		7	12.8 ± 3.8	
ER status			0.96			0.35
Negative	25	2.6 ± 0.7		87	3.9 ± 1.8	
Positive	44	2.5 ± 0.5		139	7.9 ± 2.5	
PR status			0.54			0.09
Negative	34	2.8 ± 0.7		48	3.6 ± 1.1	
Positive	35	2.3 ± 0.5		42	10.1 ± 3.7	
Her-2 status			0.28			0.54
Negative	43	2.2 ± 0.5		57	8.1 ± 3.3	
Positive	21	3.3 ± 1.0		28	5.3 ± 2.6	
Chemotherapy			0.21			0.18
Non- treatment	21	0.9 ± 0.2		26	14.4 ± 5.5	
Post-treatment	33	2.8 ± 1.0		59	5.8 ± 1.4	
Pre-& Post-treatment	1	2.2 ± 0.5		5	2.6 ± 0.2	
ND	19	3.1 ± 1.0		4	3.7 ± 0.5	
Radiotherapy			0.72			0.18
Non-treatment	38	1.6 ± 0.3		45	7.6 ± 3.5	
Post-treatment	19	3.8 ± 1.1		39	7.0 ± 2.1	
ND	17	2.1 ± 0.2		10	2.7 ± 0.6	
Tamoxifen			0.14			0.44
Non-treatment	31	1.8 ± 0.3		34	5.7 ± 1.6	
Post treatment	23	3.6 ± 1.8		27	1.3 ± 0.7	
ND	20	2.5 ± 1.2		33	2.1 ± 0.8	
Herceptin			0.85			0.45
Non-treatment	44	2.3 ± 0.7		51	9.7 ± 0.3	
Post treatment	16	2.7 ± 0.8		27	6.0 ± 1.5	
Pre- & Post-treatment	4	2.8 ± 0.3		3	6.8 ± 1.3	
ND	9	2.6 ± 1.8		13	5.6 ± 1.5	

Fold ratios of PPM1F1 mRNA expression were determined in normal/tumor or tumor/normal paired samples. Data were analyzed using either the Independent T-test (ER, PR, Her-2, and Age groups) or by One-Way ANOVA analyses for the other groups.

* A *p*-value < 0.05 was considered as statistically significant. All *p-*values are two-sided.

§ mean: average fold ratio of PPM1F1 mRNA expression in each group.

We also determined the clinical status of each patient, in order to ascertain whether higher PPM1F mRNA expression in advanced-stage tumors is important for clinical/therapeutic outcomes (Table 1). Higher PPM1F mRNA expression (T>N) correlated positively with tumor size and stage (*p = 0.01 and *p = 0.02, respectively, Table 1, and Supplementary Figure 1). Higher PPM1F expression in tumor tissue (T>N) was also associated with smoking status in these patients (Table 1, *p = 0.02). As shown in Figure 1F, *PPM1F* mRNA expression was greater in tumor tissues from active smokers (n=7) than in those from non-smokers (n=28) (bar 6 vs. bar 4; 12.8-fold vs. 6.3-fold, difference = 6.5-fold, 95% CI = 2.5 to 10.5-fold, *p = 0.01). *PPM1F* mRNA expression was also greater in tumor tissues from passive smokers (n=30) than in those from non-smokers (n=28), although the fold increase was not as large as that for active smokers (bar 5 vs. bar 4;10.3-fold vs. 6.3-fold, difference = 4.0-fold, 95% CI = 2.4 to 5.6-fold, *p = 0.026).

### Nicotine treatment induced PPM1F protein expression in breast cancer cells

**Figure 2:**
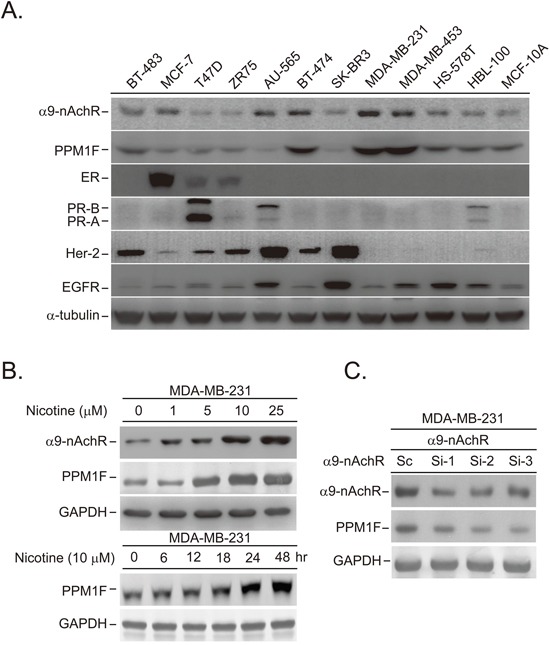
Nicotine-induced PPM1F expression was detected in breast cancer cells **A.** Detection of PPM1F protein expression by Western blotting in normal and cancerous human breast cell lines. **B.** MDA-MB-231 cells were treated with nicotine in a time (10 μM for 6-48 hours)- and dose (1-25 μM, 24 hours)-dependent manner. Immunoblotting analysis was performed, and the levels of α9-nAChR and PPM1F were determined. **C.** The expression of PPM1F protein was detected by Western blotting in the α9-nAChR knockdown MDA-MB-231 cells that were established in our previous study [21].

To investigate the protein expression profiles of PPM1F and α9-nAChR in human breast cancer cell lines, we performed Western blotting on 10 breast cancer cell lines (BT474, AU565, SKBR3, ZR75, BT483, T47D, MDA-MB-231, MCF-7, MDA-MB-453 and HS578T) and two normal breast epithelial cell lines (HBL100, MCF-10A) (Figure 2A). PPM1F expression was higher in breast cancer cell lines (BT474, MDA-MB-231, MDA-MB-453) than in normal breast cell lines (HBL100 and MCF-10A) (Figure 2A and Supplementary Figure 2).

We previously demonstrated that α9-nAChR was overexpressed in smoking-induced breast tumor tissues [21, 29]. These results implied that the elevated mRNA levels of *PPM1F* in active smokers may be due to the long-term exposure of α9-nAChR-expressing cells to nicotine. Thus, the MDA-MB-231 cell line was selected and treated with various doses of nicotine (1-25 μM) for various lengths of time (6-48 hours) (Figure 2B). PPM1F expression was induced after cells were treated with at least 5 μM nicotine for 24 hours. The nicotine-induced upregulation of PPM1F was detected as early as 18 hours in the MDA-MB-231 cells treated with 10 μM nicotine (Figure 2B).

As shown in Figure 2B, the induction of PPM1F concurred with the induction of α9-nAChR in nicotine-treated MDA-MB-231 cells, implying that PPM1F expression might increase through the nicotine-enhanced α9-nAChR-induced signaling pathway. To test this possibility, we evaluated the expression of PPM1F in three stable α9-nAChR-knockdown cell lines [21] (Figure 2C). PPM1F protein levels were lower in stable α9-nAChR knockdown MDA-MB-231 cells than in control cells (Figure 2C). These results suggested that α9-nAChR-induced PPM1F RNA expression promotes smoking-induced breast cancer formation, as observed in clinical samples (Figure 1F).

**Figure 3:**
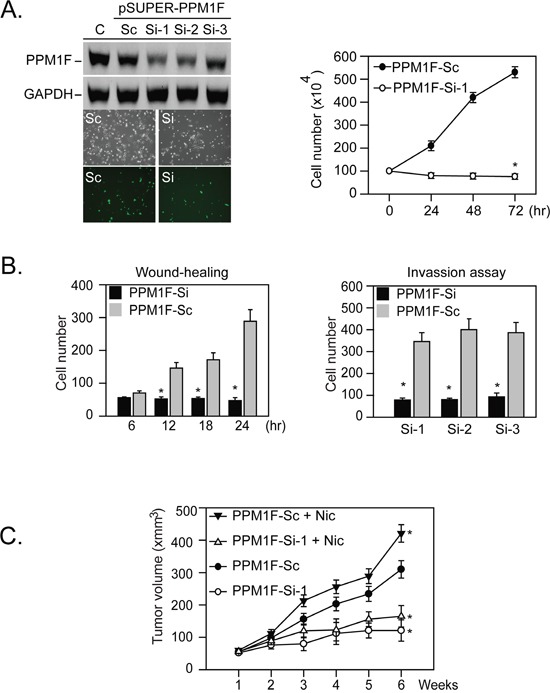
Knockdown of PPM1F expression inhibits human breast cancer cell proliferation, migration, and *in vivo* tumor growth **A.** Right panel, cell proliferation assays were performed with wild-type MDA-MB-231 cells transiently expressing PPM1F-Si1, or a scrambled (PPM1F-Sc) control plasmid (*p = 0.001). Left panel, to avoid transient plasmid DNA transfection-induced cytotoxicity, stable PPM1F-siRNA- or PPM1F-Sc-expressing MDA-MB-231 clones were established by the G418 selection method. The PPM1F levels in the selected clones were confirmed by Western blotting. **B.** Wound healing migration and invasion assays were performed with MDA-MB-231 cells stably expressing PPM1F-Si or PPM1F-Sc for 24 hours (*p = 0.001). **C.** Effect of PPM1F knockdown on nicotine-induced tumorigenesis by MDA-MB-231 cells in nude mice. PPM1F-Si or PPM1F-Sc cells (5 × 10^6^) were injected subcutaneously into the back of each NOD-SCID mouse (n = 5). After tumor transplantation, nicotine (10 mg/mL) was administered via the drinking water for six weeks until the mice were killed. The gross appearance of the tumors was then observed, and the tumor volumes were analyzed as described in the Materials and Methods [21].

To test this hypothesis, we inhibited PPM1F protein by transfecting cells with PPM1F-siRNA plasmids. Transient transfection with PPM1F-SiRNA plasmid significantly inhibited tumor cell growth (Figure 3A, right panel, *p = 0.001). We then established stable PPM1F knockdown cell lines (denoted as PPM1F-Si1, -Si2 and -Si3; Figure 3A, left panel). Stable expression of these PPM1F-Si plasmids significantly reduced the migratory and invasive capabilities of MDA-MB-231 cells (Figure 3B).

In addition, MDA-MB-231 cells stably expressing PPM1F-Si or PPM1F-Sc were introduced as xenografts in severe combined immunodeficiency (SCID) mice. Stable expression of PPM1F-siRNA in MDA-MB-231 cells attenuated nicotine-stimulated proliferation and growth and reduced the tumor volumes (Figure 3C, n = 5 mice per group; mean tumor volume at treatment week 6 in mice injected with PPM1F-Si cells = 799.3 mm^3^, in mice injected with parental cells = 2920.2 mm^3^, difference = 2120.9 mm^3^, 95% CI = 1720 to 2521.8 mm^3^, *p = 0.005). All these results suggested that PPM1F is involved in breast tumor formation.

**Figure 4:**
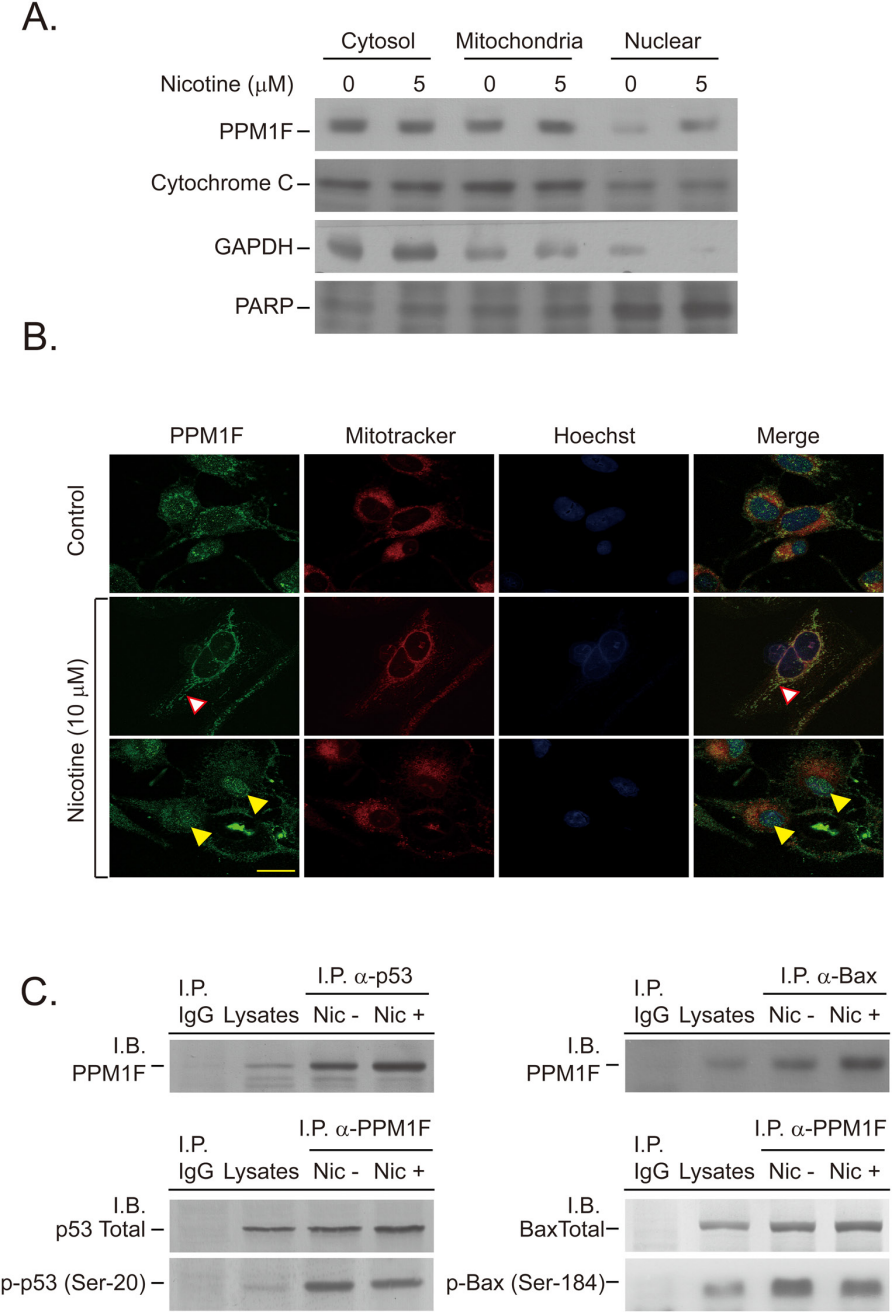
Nicotine induces PPM1F nuclear and mitochondrial translocation and complex formation with p53 and BAX **A.** MDA-MB-231 cells were treated with nicotine (5 μM) for 24 hours. The nuclear and mitochondrial lysates were isolated according to the manufacturer's instructions, and the protein levels of PPM1F and markers such as PARP, cytochrome C and GAPDH were determined by Western blotting of equal amounts of protein. **B.** MDA-MB-231 cells were stained for PPM1F (FITC-labeled), a mitochondrion-specific marker (MitoTracker), and Hoechst dye to determine nuclear localization. The intracellular localization of PPM1F was observed by confocal microscopy, as described in the Materials and Methods. (Scale bar = 25 μm) **C.** MDA-MB-231 cells were treated with or without nicotine (10 μM) for 24 hours (lanes 3-4). Cells subjected to various treatment regimens were assessed for the presence of protein-protein interactions among PPM1F, p53 and BAX. For each sample, PPM1F-associated p-p53 (Ser-20) or p-BAX (Ser-184) was immunoprecipitated (I.P.) from 200 μg of lysate as described above, and the presence of PPM1F, p53, p-p53 (Ser-20), BAX, or p-BAX (Ser-184) in the immunoprecipitated material was determined by Western blotting (or immunoblotting, I.B.) with specific antibodies.

The results described above revealed that PPM1F protein is expressed concomitantly with α9-nAChR and is induced in breast cancer cells in response to nicotine treatment (Figure 2B). To explore this in greater detail, we treated cells with nicotine, and performed Western blotting to determine whether PPM1F localized to the cytosolic, mitochondrial, or nuclear fraction (Figure 4A). PPM1F protein expression was significantly induced by nicotine (5 μM, for 24 hour), and the protein localized to the cytosolic fraction of MDA-MB-231 cells (Figure 4A, lane 1 vs. 2). However, PPM1F expression was also elevated in the mitochondrial and nuclear fractions in response to nicotine (Figure 4A, lane 3 vs. 4 and lane 5 vs. 6). To further confirm this evidence, we performed immunofluorescence staining. After nicotine treatment (10 μM for 24 hours), PPM1F translocated to the mitochondria, where it co-localized with MitoTracker (Figure 4B, indicated with a white arrowhead). Under the same treatment conditions, nicotine also induced PPM1F translocation to the nucleus, where it was observed by confocal microscopy and found to co-localize with the nuclear-staining Hoechst dye (Figure 4B, indicated with a yellow arrowhead).

Intracellular stress, such as DNA damage or other cytotoxic effects, activates genes that respond to DNA damage or other genomic aberrations. For example, DNA damage induces the phosphorylation of p53 at Ser-15, Ser-20 and Ser-37, thereby weakening its binding to the oncogenic protein MDM2, which would otherwise promote the ubiquitination and proteasomal degradation of p53 [13]. Immunoprecipitation was performed to test whether PPM1F and p53 would form a complex after nicotine treatment. Indeed, nicotine exposure increased the complex formation between PPM1F and p53 (Figure 4C, left). Subsequently, the active form, p-p53 (Ser-20), was dephosphorylated by PPM1F, and the complex formation between PPM1F and p-p53 (Ser-20) decreased (Figure 4C, left). These results suggested that the nicotine-induced translocation of PPM1F to the nucleus triggers binding between PPM1F and p-p53 (Ser-20), resulting in dephosphorylation of the active p-p53 (Ser-20). The dephosphorylated p53 can then be degraded by MDM2-induced mechanisms, resulting in nicotine-induced cell survival and tumorigenesis.

To test whether the nicotine-induced mitochondrial translocation of PPM1F (Figure 4A and 4B) allows PPM1F to bind to and dephosphorylate pro-apoptotic proteins (such as BAX), immunoprecipitation was performed to evaluate the complex formation between PPM1F and the active form of BAX, p-BAX (Ser-184). Complex formation between PPM1F and total BAX protein increased in nicotine-treated cells (Figure 4C, upper right). However, the complex formation between PPM1F and p-BAX (Ser-184) decreased in the nicotine-treated cells (Figure 4C, right). These results suggested that the nicotine-induced translocation of PPM1F to the mitochondria triggers the binding of PPM1F to p-BAX (Ser-184), which then promotes p-BAX (Ser-184) dephosphorylation and attenuates BAX-induced apoptosis. All these results suggested that the nicotine-induced translocation of PPM1F to either the mitochondria or the nucleus inactivates pro-apoptotic proteins (such as p53 and BAX) and thus stimulates oncogenesis.

**Figure 5:**
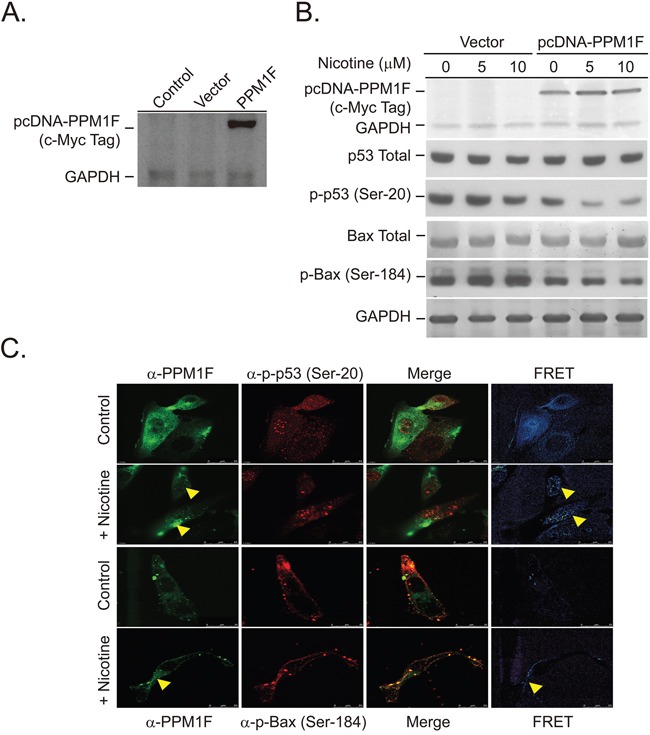
Overexpression of PPM1F in the nucleus and mitochondria induces dephosphorylation of p53 and BAX **A.** We established *PPM1F*-overexpressing MDA-MB-231 cells by transfecting cells with the pcDNA3.1 plasmid and selecting with hygromycin B, as described in the Materials and Methods. **B.** *PPM1F*-overexpressing cells and vector control cells were treated with or without nicotine (5-10 μM). The total p53, p-p53 (Ser-20), total BAX, and p-BAX (Ser-184) levels were detected by Western blotting. **C.** MDA-MB-231 cells were treated with nicotine (10 μM) for 24 hours. The intracellular interactions between PPM1F and p-BAX (Ser-184) or PPM1F and p-p53 (Ser-20) were detected based on FRET activity. The cells were stained with anti-PPM1F (FITC-labeled), anti-p-p53 (Ser-20) (rhodamine-labeled), and anti-p-BAX (Ser-184) (rhodamine-labeled) antibodies. Confocal microscopy was performed, and FRET activity (indicated with a yellow arrow) was determined as described in the Materials and Methods. (Scale bar = 25 μm)

To test this hypothesis, we established stable *PPM1F*-overexpressing cells using a pcDNA3.1TM/myc-His vector. PPM1F was expressed as a recombinant protein along with a His Tag (6x) and a c-Myc-Epitope-Tag (Figure 5A). The recombinant PPM1F protein was detected with a monoclonal antibody specific to c-Myc-Epitope-Tag-PPM1F (Figure 5A and 5B). Both the *PPM1F*-overexpressing and vector control cells were treated with nicotine (5 and 10 μM) for 24 hours (Figure 5B). We found that p-p53 (Ser-20) and p-BAX (Ser-184) were dephosphorylated in the nicotine-treated pcDNA3.1-PPM1F cells (Figure 5B, lane 4 vs. lanes 5 and 6). In addition, even without nicotine treatment, significant dephosphorylation of p-p53 (Ser-20) and p-BAX (Ser-184) was observed in *PPM1F*-overexpressing cells relative to vector control cells (Figure 5B, lane 1 vs. lane 4). To further confirm whether p-p53 (Ser-20) and p-BAX (Ser-184) were dephosphorylated by PPM1F through direct complex formation, pcDNA3.1-PPM1F cells were treated with nicotine (10 μM, 24 hours), and fluorescence resonance energy transfer (FRET) photobleaching was performed to detect PPM1F/p-p53 (Ser-20) and PPM1F/p-BAX (Ser-184) complex formation (Figure 5C). A significant FRET signal was detected in the nicotine-treated pcDNA3.1-PPM1F cells (Figure 5C, yellow arrowhead). These results explain the mechanisms underlying the higher PPM1F levels involved in inactivation of p53-mediated apoptosis pathway in breast tumor tissues from smokers compared to non-smokers.

## DISCUSSION

We previously demonstrated that α9-nAChR activation is an important inducer of breast cancer [21]. In tumor tissues, higher levels of the α9-nAChR subunit were detected in advanced-stage breast cancer patients with poor survival outcomes when compared to the early-stage patients [21, 29]. We also found that α9-nAChR expression was higher in tumor tissues when compared to adjacent normal tissues in heavy smokers (6.8-fold) than in non-smokers (1.5-fold) [21]. We proposed that receptor-induced signaling activation is more important than chemical-induced effects in promoting smoking-induced carcinogenesis [22]. In addition, we demonstrated that nicotine induces the proliferation, migration and metastasis of colon and bladder cancer cells [30–32]. For these reasons, we proposed that blocking the receptors induced by smoking would be an easier approach than prescribing smoking cessation for the prevention of breast cancer [33].

Many kinds of natural compounds have been tested for their ability to inhibit a9-nAChR expression, and we found that inhibition of the signals induced by this receptor significantly diminished the effects of smoking [34–37]. Our previous study demonstrated that the estrogen-induced estrogen receptor is also promoted in nicotine-induced a9-nAChR activation [29]. We further demonstrated that nicotine-induced a9-nAChR protein upregulation could easily be detected in human breast cancer cells (10 μM for 24 hours), but could not be detected in normal cells (MCF-10A) [29]. These observations implied that certain important candidate genes (such as α9-nAChR) could be activated through estrogen-induced transcriptional activation.

PPM1F (also known as POPX2) is a Ser/Thr protein phosphatase that is overexpressed during breast tumor invasion [27]. The levels of PPM1F protein are higher in invasive MDA-MB-231 breast cancer cells than in noninvasive MCF7 cells [27]. We previously reported that the overexpression of α9-nAChR was associated with poor outcomes in patients with breast cancer [21, 29]. In the present study, the silencing of α9-nAChR also reduced PPM1F protein levels, ultimately reducing cell motility and invasiveness (Figures 2C, 3C). Similarly, a previous report indicated that PPM1F increased cell motility by promoting microtubule-severing activity [24]. Silencing of *PPM1F* decreased the phosphorylation of MAPK1/3 and GSK3α/β and thereby reduced the activity of these kinases. In addition, *PPM1F* overexpression enhanced NIH3T3 cell motility in scratch wound assays [28]. In this study, we found higher levels of *PPM1F* in breast tumor tissues from smokers than from non-smokers. All these results suggested that PPM1F could promote smoking-induced breast cancer.

We also found that overexpression of *PPM1F* reduced the level of phosphorylated p53 (Ser-20) and phosphorylated BAX (Ser-184) in nicotine-treated MDA-MB-231 cells. These results revealed that PPM1F is a phosphatase that inactivates pro-apoptotic proteins (such as BAX) and proteins involved in cell cycle arrest (such as p53). These results could explain why higher levels of *PPM1F* were detected in patients with advanced-stage breast cancer (stages 3-4) than in those with early-stage breast cancer (Figure 1D, *p=0.04). A previous *in vivo* study also demonstrated that PPM1F drastically increased the initial surface attachment of MDA-MB-231 cells in the lungs [27].

Few studies have investigated the specific genetic profile of the *p53* gene in breast cancer [7, 8, 38]; however, the *p53* mutation patterns in breast cancer and lung cancer are similar, and both may be initiated by environmental factors such as cigarette smoking. A case-control study demonstrated with immunohistochemistry that p53 was overexpressed in 44.4% (168/378) of the breast cancer cases in young women (under 45 years) [8]. Immunohistochemistry has been used to measure p53 protein levels directly for large-scale epidemiological research, and such studies have demonstrated a strong correlation between p53 expression and mutation [39–41]. All these results suggest that p53 protein levels and p53 accumulation in the nucleus may be associated with exposure to environmental carcinogens such as cigarette smoke in patients with aggressive forms of breast cancer [41]. Thus, smoking-induced phosphorylation and/or dephosphorylation of p53 could be a very early step of carcinogenic signaling (immediately after smoking). For this reason, p53-phosphorylation-induced mechanisms could be important for breast cancer formation, therapy, and prevention.

**Figure 6:**
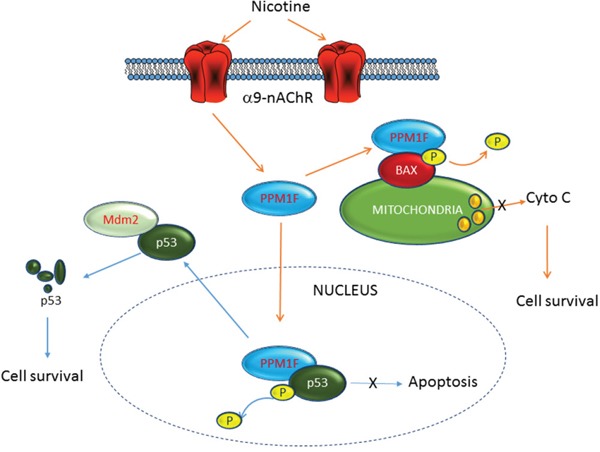
Schematic representation of the involvement of PPM1F in nicotine-induced breast cancer tumorigenesis Higher PPM1F protein levels were detected in advanced-stage breast cancer tissues, as well as breast cancer tissues from patients with a smoking history. We demonstrated that knockdown of α9-nAChR also reduced PPM1F expression. In contrast, nicotine treatment induced PPM1F expression in MDA-MB-231 cells. We found that after nicotine treatment, PPM1F triggered the inactivation of both p-p53 and p-BAX by dephosphorylating them at specific sites. These results suggest that PPM1F could be downstream of α9-nAChR in nicotine-induced breast cancer formation, and could attenuate p-p53 (Ser-20)- and p-BAX (Ser-184)-induced pro-apoptotic pathways.

As shown in Figure 1F, *PPM1F* expression was associated with smoking behavior and was higher in the patients with advanced-stage (stages 3-4) breast cancer than in those with early-stage breast cancer. Based on our study results, we propose that PPM1F functions as a phosphatase to dephosphorylate p53, thereby inactivating p53 and increasing its degradation (Figure 6). We found that PPM1F expression correlated with α9-nAChR expression, as an *in vitro* study demonstrated that nicotine induced both α9-nAChR and PPM1F expression. Our study demonstrated that inhibition of *PPM1F* with siRNA significantly diminished the dephosphorylation of p-p53, thereby increasing the p-p53 (Ser 20) level, inducing cell growth arrest, and eventually suppressing tumor growth (Figure 6). These observations indicate that PPM1F could work downstream of α9-nAchR to promote nicotine-induced carcinogenic signals. Thus, PPM1F expression could be used for prognostic diagnosis, or inhibited as a potential strategy for cancer prevention and therapy.

## MATERIALS AND METHODS

### Cell lines

Human mammary gland epithelial adenocarcinoma cell lines (luminal subtype A: BT474 (ATCC HTB-20), BT483 (ATCC HTB-121), MCF-7 (ATCC HTB-22), T47D (ATCC HTB-133), and ZR75-1 (ATCC CRL-1500) [42]; luminal subtype B: AU565 (ATCC CRL-2351), MDA-MB-453 (ATCC HTB-131), and SKBR3 (ATCC HTB-30) [43–45]; basal subtype: MDA-MB-231 (ATCC HTB-26) HS578T (ATCC HTB-126) [46]); the normal human breast epithelial cell line MCF-10A (ATCC CRL-10317); and an immortalized cell line obtained from a primary culture of cells derived from an early lactation sample of human milk, HBL100 (ATCC HTB-124), were purchased from the American Type Culture Collection (ATCC, Manassas, VA, USA). MCF-10A cells were maintained in MCF-10A culture medium consisting of DMEM/F12 (Thermo Fisher Scientific, Passau, Germany) supplemented with 20 ng/mL epidermal growth factor, 10 g/mL insulin, 0.5 g/mL hydrocortisone, and 1X non-essential amino acids (Thermo Fisher Scientific). BT474, BT483, MCF-7, MDA-MB-453, MDA-MB-231, and HBL-100 cells were maintained in DMEM (Thermo Fisher Scientific). T47D, ZR-75 AU-565, SKBR3, and HS-578T cells were maintained in DMEM/F12 (Thermo Fisher Scientific). The cells were cultured according to standard protocols [21].

### Cell proliferation and viability assays

Cell growth and proliferation were determined by the 3-(4,5-dimethylthiazol-2-yl)-2,5-diphenyltetrazolium (MTT) assay [47]. This assay was repeated four times with duplicate samples.

### RNA interference

*PPM1F* expression was ablated in MDA-MB-231 breast cancer cells by means of at least two independent siRNA clones. Scrambled sequences of each siRNA were used as controls. After Basic Local Alignment Search Tool (BLAST) analysis to verify the absence of significant sequence homology with other human genes, the primer sequences were designed as follows: *PPM1F* siRNA-1 forward primer: 5′-GATCCCCGAGCCCTCAGAGAAGCCTTTTCAAGAGAAAGGCTTCTCTGAGGGCTCTTTTTA-3′; *PPM1F* siRNA-1 reverse primer: 5′-AGCTTAAAAAGAGCCCTCAGAGAAGCCTTTCTCTTGAAAAGGCTTCTCTGAGGGCTCGGG-3′; *PPM1F* siRNA-2 forward primer: 5′-GATCCCCATCCGGAACACTCGCCGCATTCAAGAGATGCGGCGAGTGTTCCGGATTTTTTA-3′; *PPM1F* siRNA-2 reverse primer: 5′-AGCTTAAAAAATCCGGAACACTCGCCGCATCTCTTGAATGCGGCGAGTGTTCCGGATGGG-3′; *PPM1F* siRNA-3 forward primer: 5′-GATCCCCGGGATGTCTTCCAGAAGCCTTCAAGAGAGGCTTCTGGAAGACATCCCTTTTTA-3′; *PPM1F* siRNA-3 reverse primer: 5′-AGCTTAAAAAGGGATGTCTTCCAGAAGCCTCTCTTGAAGGCTTCTGGAAGACATCCCGGG-3′; *PPM1F* scRNA-1 forward primer: 5′-GATCCCCAGGGACAGACACTCGCTCGTTCAAGAGACGAGCGAGTGTCTGTCCCTTTTTTA-3′; *PPM1F* scRNA-1 reverse primer: 5′-AGCTTAAAAAAGGGACAGACACTCGCTCGTCTCTTGAACGAGCGAGTGTCTGTCCCTGGG-3′. The selected sequences (underline means the restriction enzyme cutting site and the bold form indicated the silencing sites) were inserted into the pSUPER vector (Oligoengine Co., Seattle, WA, USA) and digested with *Bgl*II and *Hin*dIII (underlined sections of the primer sequences) to generate the pSUPER-PPM1F-Si and pSUPER-scrambled vectors. The sequences of all the constructs were confirmed by DNA sequencing. The transfection protocol has been described previously [21].

### Generation of stable *PPM1F* siRNA-expressing cell lines

At least three clones of MDA-MB-231 cells that stably expressed *PPM1F* siRNA or scrambled control siRNA were generated (PPM1F-Si1, Si2, and Si3). The pSUPER-PPM1F-Si and pSUPER-scrambled vectors were transfected into the cells, and stable integrants were selected 72 hours later with G418 (4 mg/mL). After 30 days in selective medium, three G418-resistant clones were isolated; *PPM1F* mRNA and protein expression were >80% lower in these clones than in the control clones (scrambled control: PPM1F-Sc).

### Stable cells transfected with overexpression plasmids

A PCR fragment encompassing the coding region of the *PPM1F* gene was generated by means of the forward primer 5′-TTCAAGCTTATGTCCTCTGGAGCCCCACAG-3′, which was designed to include a *Hin*dIII site, and the reverse primer 5′-TTAGGATCCCTAGCTTCTTGGTGGAGCCTG-3′, which contained a *Bam*HI site. After digestion with *Bam*HI and *Hin*dIII, the fragment was ligated into the pcDNA3.1™/myc-His vector (Cat No. V80020; Invitrogen Co., CA, USA). PPM1F was expressed along with a His Tag (6x) and a c-Myc-Epitope-Tag. The recombinant PPM1F protein was detected with an antibody specific to the c-Myc-Epitope-Tag (R950-25, Invitrogen Co., CA, USA). Thirty days later, hygromycin B (100 μg/mL) selection was used to establish MDA-MB-231 cells with stable integration of pcDNA3.1 and pcDNA3.1-PPM1F (pcDNA3.1-Vector and pcDNA3.1-PPM1F cells) [21].

### Protein extraction, western blotting, and antibodies

To examine protein expression, we harvested human breast tumor cells for Western blotting analysis as previously described [21]. The following antibodies were purchased from Cell Signaling Technology (Beverly, MA, USA): anti-EGFR (#9922) and anti-BAX (#2772). The following antibodies were purchased from Santa Cruz Co. (CA, USA): anti-ER-α (sc-543), anti-HER2 (sc-284), anti-PR (sc-538), anti-PARP-1/2 (sc-7150), goat anti-rabbit IgG-HRP (sc-2004), anti-GAPDH, (sc-32233), anti-phosphorylated p53 (sc-18079-R), and goat anti-mouse IgG-HRP (sc-2005). The following antibodies were purchased from Abcam Inc. (Cambridge, MA): anti-α-tubulin (ab4074), anti-α9-nAChR, anti-p53 [DO-1] (ab1101), anti-phosphorylated BAX (Ser-184) (ab111391), anti-PPM1F (ab156222) and anti-cytochrome C [7H8.2C12] (ab13575).

### Wound healing cell migration and *in vitro* invasion assay

MDA-MB-231 cells expressing PPM1F-Si1 or PPM1F-Sc were seeded into six-well plates for *in vitro* wound healing migration assays according to a previously reported protocol [29]. *In vitro* invasion assays were performed in 10-mm Transwell chambers that contained Matrigel-coated polycarbonate membranes with 8-μm pores (Corning Costar, Cambridge, MA, USA), as previously described [30]. MDA-MB-231 cells expressing PPM1F-Si1 or PPM1F-Sc plasmids were trypsinized and suspended at a final concentration of 5 × 10^5^ cells/mL in serum-free L15 medium. The cell suspensions were then added to the upper Transwell chambers. The bottom chambers contained medium with 5% FBS as a chemoattractant. After a 24-hour incubation at 37°C in 5% CO_2_ and 95% air, all the non-invading cells were removed from the upper surface of the Transwell membrane with a cotton swab. The invading cells were fixed with 100% methanol, stained with hematoxylin and eosin (Nanjing Sunshine Biotechnology Ltd., Nanjing, China), and counted under a microscope. Ten fields were counted for each assay.

### Patient samples

The participants in this study (n = 167) provided their written informed consent. The study and consent procedures were approved by the Research Ethics Committee of Taipei Medical University Hospital. Pairs of human breast tumor and adjacent normal epithelial tissues were obtained from anonymous donors according to a protocol that was approved by the institutional review board (TMU-JIRB, No. 201407014). All the clinical investigations were conducted according to the principles expressed in the Declaration of Helsinki. Histological inspections revealed that all the patient samples consisted of more than 80% tumor tissue. All the samples (paired tumors and normal tissues) were collected and categorized according to their clinical characteristics.

### Animal experiments

MDA-MB-231 cell lines with stable integration of pSUPER-PPM1F-Si or pSUPER-PPM1F-Sc sequences were established by G418 selection. The cells (5 × 10^6^) were implanted subcutaneously into each six-week-old NOD.CB17-PRKDC(SCID)/J(NOD-SCID) mouse (n = 5) (purchased from the National Science Council Animal Center, Taipei). After tumor transplantation, nicotine (10 mg/mL) was administered via the drinking water for six weeks [21] until the mice were killed by anesthesia with ether. During the experiment, the tumor size was measured with calipers and the tumor volume was estimated with the following formula: tumor volume (mm^3^) = 1/2 x *L* x *W*^2^, where *L* is the length and *W* is the width of the tumor [21]. At the end of the experiment, subcutaneous tumor masses were dissected from the mice and weighed. All mouse protocols were performed according to an Association for Assessment and Accreditation of Laboratory Animal Care (AALAC)-approved protocol.

### RNA isolation and quantitative real-time PCR

Total RNA from human breast tumors and normal tissue samples acquired directly from patients was isolated with TRIzol (Invitrogen, Carlsbad, CA, USA) according to the manufacturer's protocol [21]. The *PPM1F*-specific PCR primers were synthesized as Forward: 5′-GCCTACTTTGCTGTGTTTGA-3′ and Reverse: 5′-TCTCGCTTGGCTTTCCT-3′, and the β-glucuronidase-specific primers were synthesized as Forward: 5′-AGTGTTCCCTGCTAGAATAGATG-3′ and Reverse: 5′-AAACAGCCCGTTTACTTGAG-3′. A LightCycler thermocycler (Roche Molecular Biochemicals, Mannheim, Germany) was used for quantitative real-time PCR. The *PPM1F* mRNA fluorescence intensity was measured and normalized to glucuronidase expression through the built-in software (Roche LightCycler Version 4) [21].

### Antibodies, immunohistochemistry and microscopic observation

The localization of PPM1F protein in breast tumor tissues was detected by immunohistochemistry [21]. The sections were microwaved in Tris buffer (pH 6) for 10 min. Then, the sections were blocked in 5% horse serum (Chemicon, Temecula, CA, USA) for 30 min and subsequently incubated with a 1:100 dilution of an antibody targeting PPM1F (Cat. No. H00002517-M01, clone 1D4; Abnova Co., Atlanta, GA, USA) for 2 hours at room temperature. After the sections were incubated with the primary antibody, they were stained with an LSAB 2 kit (Dako, Carpinteria, CA, USA), which employs a streptavidin-biotin-peroxidase method. Then, the slides were washed, dehydrated and coverslipped with DPX (Sigma-Aldrich, St. Louis, MO, USA). Adjacent sections and slides were counterstained with hematoxylin for general histological evaluation.

### Confocal microscopy, immunofluorescence and FRET photobleaching

An immunofluorescence assay was performed to investigate whether PPM1F and BAX Ser-184 (or p53 Ser-20) would form complex in the human MDA-MB-231 breast cancer cell line, and images were subsequently captured via confocal microscopy [48]. This procedure was followed by incubation with 1:100 diluted primary monoclonal antibodies (anti-PPM1F conjugated to FITC or anti-BAX Ser-184 conjugated to rhodamine) in blocking solution for 1 hour at room temperature. The samples were washed three times with PBS and mounted with Gel Mount (Sigma), and images were acquired with a Leica TCS SP5 Confocal Spectral Microscope Imaging System (Leica Microsystems, Wetzlar, Germany) as described previously [36]. The acceptor bleaching FRET method [48, 49] was used according to the manufacturer's instructions (FRET Wizards in the Leica Application suite). Briefly, the initial donor (FITC 488) image represents donor fluorescence in the presence of the acceptor, rhodamine. After complete photobleaching of the acceptor, a second donor image was collected. Quantitative analysis of the D/D_A_ values was performed according to the method of Xia and Liu [49] after image acquisition with Leica Application Suite-Advanced Fluorescence software (Leica LAS AF).

### Immunoprecipitation assay

The possible association of PPM1F with phosphorylated p53 (Ser-20) and phosphorylated BAX (Ser-184) was evaluated through immunoprecipitation. Briefly, the p-53-, PPM1F- or p-BAX-associated proteins were precipitated from 200 μg of protein lysate from each sample by means of anti-p-p53, anti-p-BAX or anti-PPM1F antibodies (2 μg) and protein A-agarose beads (20 μL) according to standard procedures. The immunoprecipitated proteins were then electrophoresed by 12% SDS-PAGE, and p53 (total), p-p53 (Ser-20), BAX (total), p-BAX (Ser-184), and PPM1F were evaluated by Western blotting. The gel was dried and subjected to autoradiography.

### Statistical methods

All the data are expressed as the mean of at least three experiments with 95% confidence intervals (CIs) unless otherwise stated. A paired t-test was used to compare *PPM1F* mRNA expression in paired normal and tumor tissues from patients with breast cancer. The fold ratios of *PPM1F* mRNA expression in tumors vs. normal samples were compared through the Mann-Whitney U test. Pearson correlation coefficient tests were used to identify associations between PPM1F protein expression and clinico-pathological variables. The endpoint was overall survival, which was calculated from the date of surgery to the final follow-up date. The median length of follow-up was 60 months (range, 2-152 months). Survival analyses were performed with the Cox proportional hazards model. Survival curves were plotted with the Kaplan-Meier method, and log-rank tests were performed to evaluate prognostic differences between groups for categorical variables. All the statistical comparisons were performed with SigmaPlot graphing software (San Jose, CA, USA) and Statistical Package for the Social Sciences, v. 16.0 (SPSS, Chicago, IL, USA). All the statistical tests were two-sided. A p-value of 0.05 or less was considered statistically significant.

## SUPPLEMENTARY MATERIALS FIGURES


