# Supplementary material for: Protein phosphatase Mg2+/Mn2+ dependent 1F promotes smoking-induced breast cancer by inactivating phosphorylated-p53-induced signals
Source: Oncotarget. 2016 Oct 18;7(47):77516–31. doi: 10.18632/oncotarget.12717 (PMC5363601; doi:10.18632/oncotarget.12717)
Supplement: Supplementary file 1 [file oncotarget-07-77516-s001.pdf]

Protein phosphatase Mg2+/Mn2+ dependent 1F promotes smoking-induced breast cancer by inactivating phosphorylated-p53-induced signals

SUPPLEMENTARY FIGURES

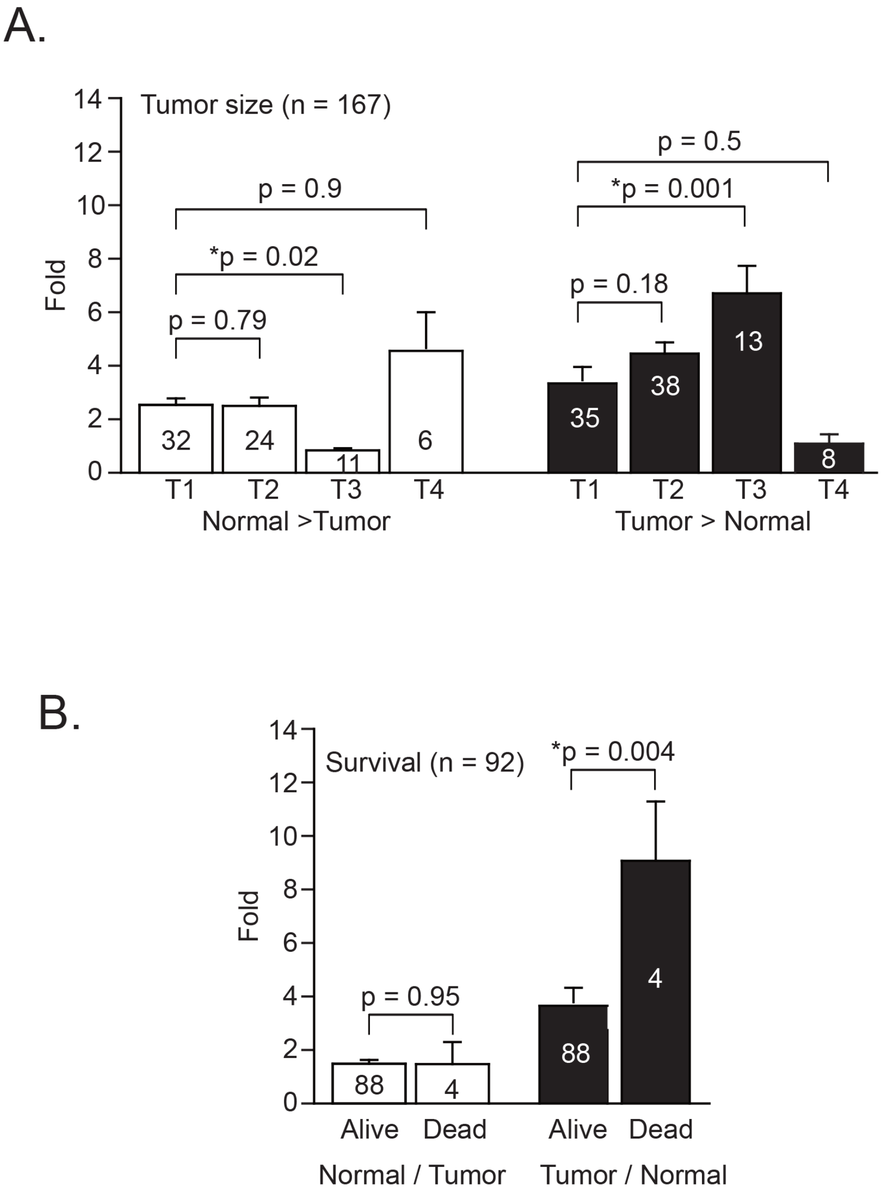

Supplementary Figure S1: *PPM1F* mRNA levels in human breast tumor tissues. *PPM1F* mRNA expression profiles in paired human breast tumor (red lines) and normal (green lines) tissues (n = 167) were evaluated by real-time PCR and calculated according to clinico-pathological factors. **A.** *PPM1F* mRNA levels were associated with tumor size (T1 to T4). The data were analyzed with an overall nonparametric test (Kruskal-Wallis test), and multiple comparisons were performed with the Mann-Whitney test. All the p-values are two-sided. **B.** *PPM1F* mRNA levels were calculated and associated with survival. *PPM1F* mRNA levels were higher in samples from four patients who died than in those from 88 patients who survived. (* p=0.004)

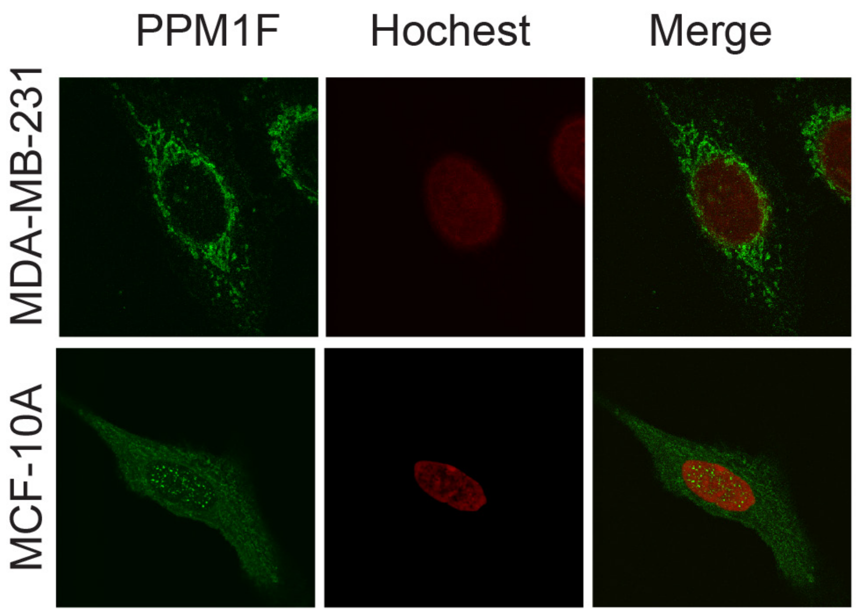

Supplementary Figure S2: PPM1F protein expression in human breast cancer and normal cells. MDA-MB-231 and MCF-10A cells were stained for PPM1F (FITC-labeled), and Hoechst dye was used to determine nuclear localization. The intracellular localization of PPM1F was determined by confocal microscopy, as described in the Materials and Methods.
